# Limited evidence of declining growth among moisture-limited black and white spruce in interior Alaska

**DOI:** 10.1038/s41598-017-15644-7

**Published:** 2017-11-10

**Authors:** Patrick F. Sullivan, Robert R. Pattison, Annalis H. Brownlee, Sean M. P. Cahoon, Teresa N. Hollingsworth

**Affiliations:** 1 Environment and Natural Resources Institute, University of Alaska Anchorage, Anchorage, AK 99508 USA; 2 Pacific Northwest Research Station, USDA Forest Service, Anchorage, AK 99503 USA; 3 Boreal Cooperative Research Unit, USDA Forest Service, Fairbanks, AK 99775 USA

## Abstract

Boreal forests play critical roles in global carbon, water and energy cycles. Recent studies suggest drought is causing a decline in boreal spruce growth, leading to predictions of widespread mortality and a shift in dominant vegetation type in interior Alaska. We took advantage of a large set of tree cores collected from random locations across a vast area of interior Alaska to examine long-term trends in carbon isotope discrimination and growth of black and white spruce. Our results confirm that growth of both species is sensitive to moisture availability, yet show limited evidence of declining growth in recent decades. These findings contrast with many earlier tree-ring studies, but agree with dynamic global vegetation model projections. We hypothesize that rising atmospheric [CO_2_] and/or changes in biomass allocation may have compensated for increasing evaporative demand, leaving recent radial growth near the long-term mean. Our results highlight the need for more detailed studies of tree physiological and growth responses to changing climate and atmospheric [CO_2_] in the boreal forest.

## Introduction

The boreal forest is a critical component of global carbon, water and energy cycles^1^. However, recent studies point to increasing wildfire^2^, decreasing landscape greenness^3,4^, greater tree mortality^5^ and declining spruce growth^6–11^. Over the last two decades, numerous tree-ring studies have been completed in interior Alaska, with most concluding that declining growth of black (*Picea mariana*) and white spruce (*Picea glauca*) is a result of drought stress, induced by rising air temperature and increasing evaporative demand^6–15^. The evidence for drought stress is derived from negative correlations between growing season air temperature and growth, positive correlations between precipitation and growth and decreasing carbon isotope discrimination in tree-rings. The common conclusion that growth of black and white spruce is declining in interior Alaska as a result of temperature-induced drought stress has led some investigators to predict widespread spruce mortality and suggest that coniferous forests of interior Alaska are in the midst of a transition to temperate forests and/or grasslands^8,11^.

There is a wide range of variation in the degree to which plants regulate water loss through stomatal closure under warm and dry conditions^16^. At one end of the spectrum are anisohydric species, which exhibit relatively little stomatal regulation and allow leaf water potential to decline with soil water potential under drought. Anisohydric species tend to occupy drier habitats and have xylem conduits that are more resistant to cavitation under low water potential. At the other end of the spectrum are isohydric species, which have xylem conduits that are more vulnerable to cavitation, display strong stomatal regulation of water loss and typically maintain leaf water potential within a limited range under drought. Drought-induced mortality of plants at the anisohydric end of the spectrum is more likely to occur as a result of hydraulic failure. Meanwhile, isohydric species are thought to be more prone to mortality related to reduced carbohydrate availability, as a result of prolonged stomatal closure^17^. Carbohydrates are essential for phloem transport, turgor maintenance, re-filling of embolized xylem and production of defensive compounds. Depletion of carbohydrate reserves by maintenance respiration during extended periods of stomatal closure could lead to mortality as a result of any of the above physiological mechanisms alone or in combination with attack by insects or pathogens^18^.

Spruce are relatively isohydric in the sense that complete stomatal closure and loss of hydraulic conductance both occur at high xylem water potential. In white spruce, near complete stomatal closure was observed at a xylem water potential of ~−2.0 MPa^19^, while 50% loss of hydraulic conductance was detected at a xylem water potential of ~−4.0 MPa^20^. While anisohydric species may exhibit mortality in response to extreme climate events, drought-induced mortality of isohydric species is expected to occur in response to warm and dry conditions over a longer time period, during which stomatal closure constrains the supply of photosynthate and the demands of maintenance respiration eventually exhaust carbohydrate reserves^17^. Because drought-induced mortality of isohydric species often occurs slowly and involves depletion of carbohydrate reserves that fuel both maintenance respiration and growth, tree-ring studies commonly show a prolonged growth decline prior to drought-induced mortality^21–24^. Thus, identification of a climate-driven growth decline in the tree-ring record could be a useful tool to identify isohydric species that are at risk of drought-induced mortality.

**Figure 1:**
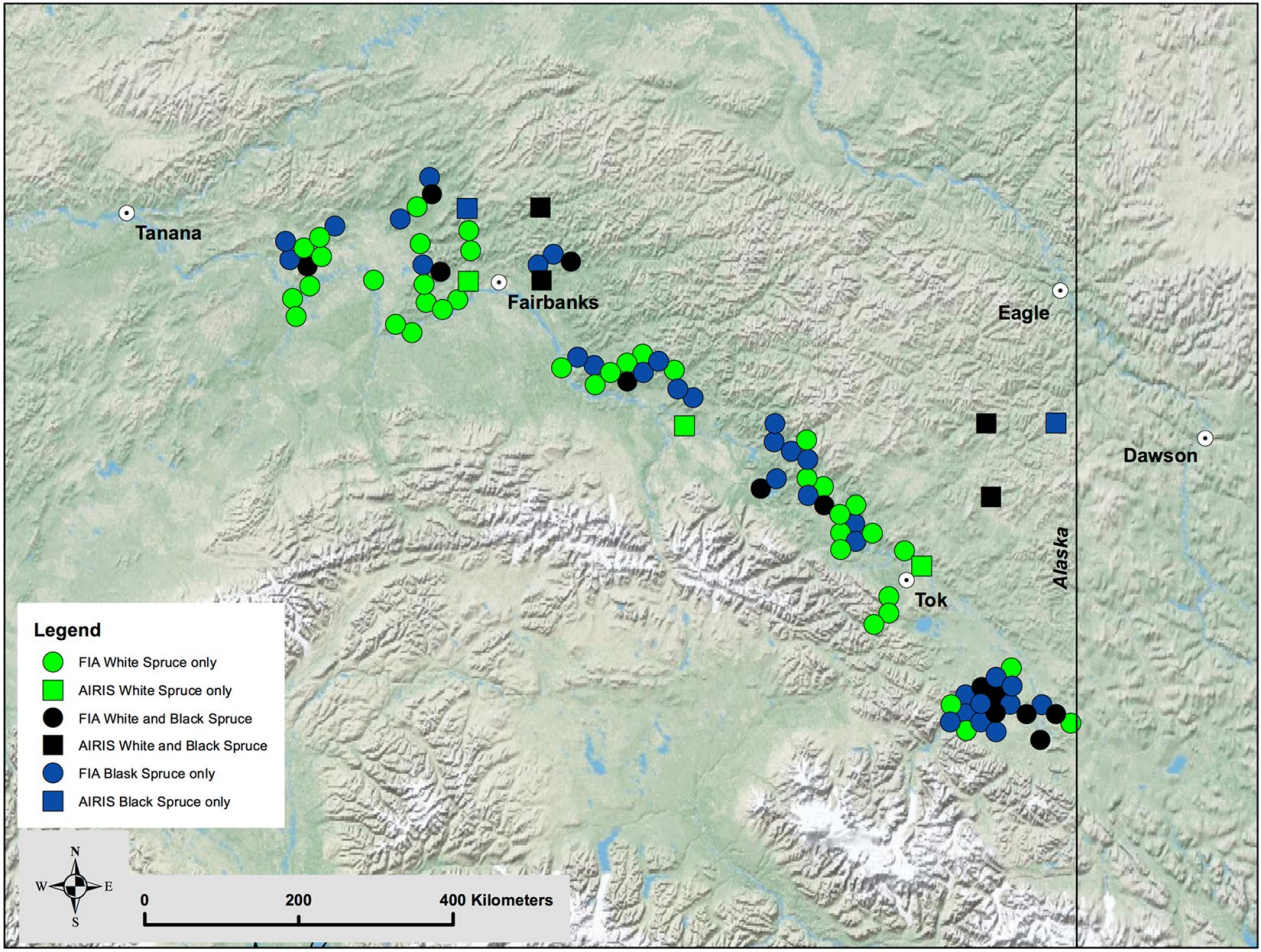
Approximate locations of the FIA and AIRIS plots where increment cores were collected in interior Alaska. Locations of Tanana, Fairbanks, Eagle, Tok and Dawson are shown for reference. The plots tend to cluster into four distinct groups, which we refer to as northwest (NW), northcentral (NC), southcentral (SC) and southeast (SE). The map was created using ArcGIS 10.4.1 (Esri, Redlands, CA). The base layer is the intellectual property of Esri and is used herein under license. Copyright © 2016 Esri and its licensors. All rights reserved.

We took advantage of a large set of tree cores collected from randomly located plots across a vast area of interior Alaska (Fig. 1) and tested many of the conclusions that have been drawn from previous tree-ring studies in the region. In an earlier study, using the same tree-ring data, we examined the effect of detrending method on apparent long-term growth trends in black and white spruce^25^. We found that choice of detrending method had important implications for apparent growth trends and the strength of climate-growth correlations. All of the methods tested revealed a pronounced peak in growth of both species centered near 1940, providing important historical context for studies that have focused on growth trends during the second half of the 20^th^ century. Of the detrending methods tested, signal-free multiple curve RCS^26^ produced the strongest and/or greatest number of significant climate-growth correlations. As observed in many previous studies, growth was negatively correlated with growing season temperature and positively correlated with August precipitation. In the present study, we carried out a more thorough evaluation of the relationships between climate and growth, used stable isotopes in tree-ring alpha-cellulose to examine potential changes in gas exchange physiology over time and analyzed a wide range of habitat variables with the aim of identifying important controls on spatial variation in recent growth of black and white spruce. Specifically, we investigated the following set of linked questions and hypotheses:

1. How have correlations between growth of black and white spruce and growing season temperature and precipitation changed over the past century? We hypothesized that temperature would have a positive influence on growth in the early part of the tree-ring records and a negative influence on growth in recent decades. We anticipated that precipitation would be weakly correlated with growth early and positively correlated with growth later in our tree-ring chronologies.
2. What are the forms (e.g., linear, sigmoidal, etc.) of the relationships between growth of black and white spruce and growing season temperature and precipitation? We hypothesized that the relationship between temperature and growth would be strongly non-linear, with a positive effect at low temperatures, a negative effect at high temperatures and a clear optimum at intermediate air temperatures. We anticipated an overall positive correlation between precipitation and growth.
3. How has the balance between photosynthesis and stomatal conductance of black and white spruce changed over the past century? In keeping with previous results, we anticipated a large decrease in carbon isotope discrimination (Δ^13^C) in tree-ring alpha-cellulose from the turn of the 20^th^ to the turn of the 21^st^ century in black and white spruce.
4. Which habitat characteristics are the most important determinants of recent growth of black and white spruce? We hypothesized that habitat characteristics that relate to moisture availability (aspect, slope, elevation, topographic position, stand density, etc.) would be the most important determinants of recent growth in both species.
5. Do black and white spruce growing in “good” and “poor” habitats show different sensitivities to climate and different long-term growth trends? We hypothesized that “poor” habitats for both species would be those in which moisture availability is expected to be most limited. Further, we anticipated that growth in “poor” habitats would be more negatively correlated with air temperature and more positively correlated with precipitation than growth in “good” habitats. Finally, we expected that tree-ring chronologies constructed separately for trees growing in “poor” habitats would show the most marked recent growth declines.

## Results

### Potential Changes in Climate-Growth Relationships over Time

**Figure 2:**
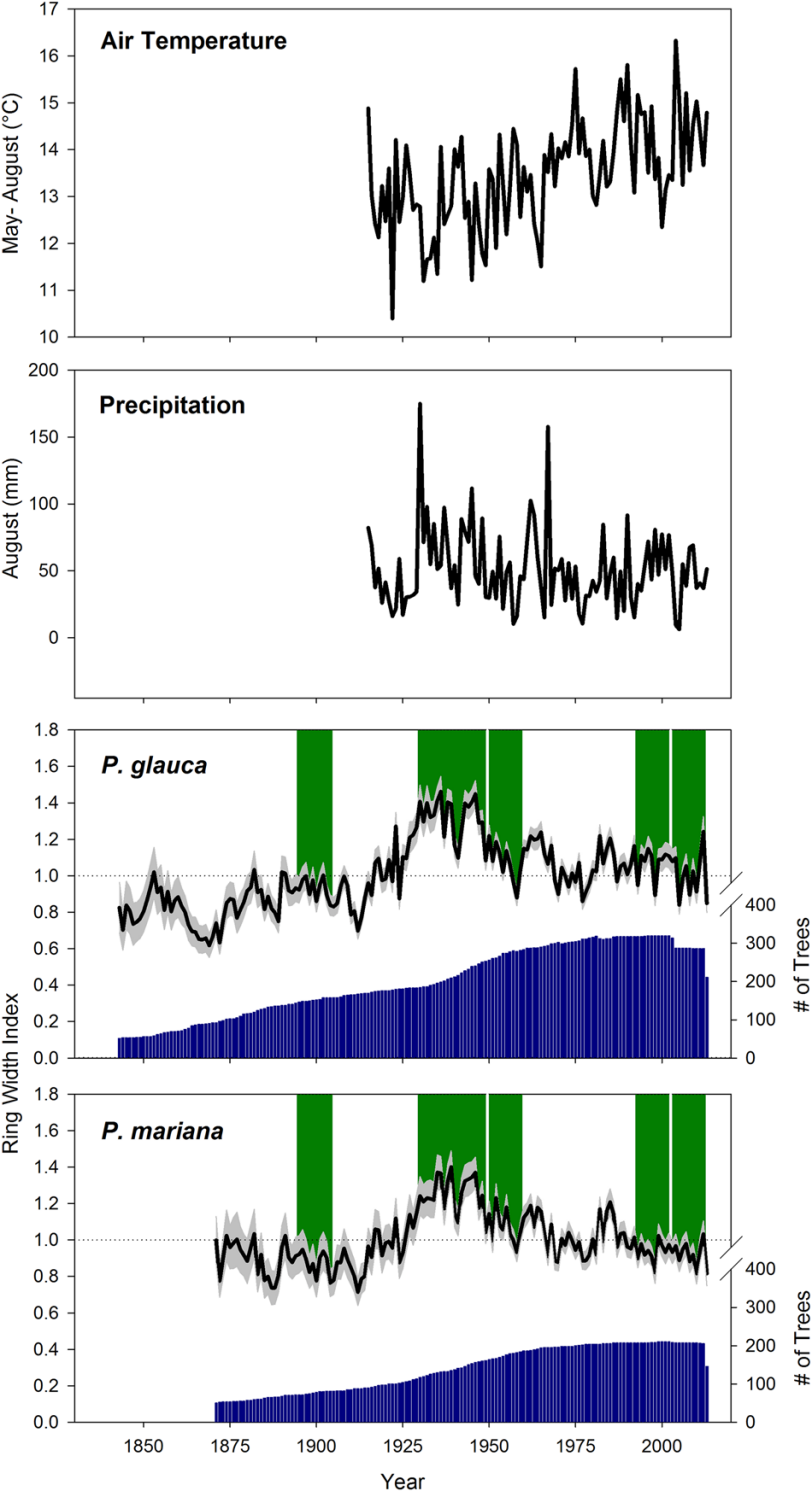
Long-term growing season air temperature and August precipitation records for Fairbanks, AK. The bottom two panels show four-curve RCS tree-ring chronologies for white and black spruce. The grey shading indicates 95% confidence intervals, the dark blue bars show variation in sample size over time, while the green shading indicates the five time periods selected for isotopic analysis. Both chronologies have a mean of 1.0 over their respective lengths.

The growing season air temperature record (May- August) for Fairbanks shows a strong warming trend that began around 1965 (Fig. 2). August precipitation, which was identified as the precipitation variable best correlated with growth of both species^25^, does not show evidence of a significant trend over time in the Fairbanks station record. However, 18 of the 50 years between 1915 and 1964 had August precipitation greater than 60 mm, while only 10 of the 50 years between 1965 and 2014 had August precipitation totals exceeding that amount.

**Figure 3:**
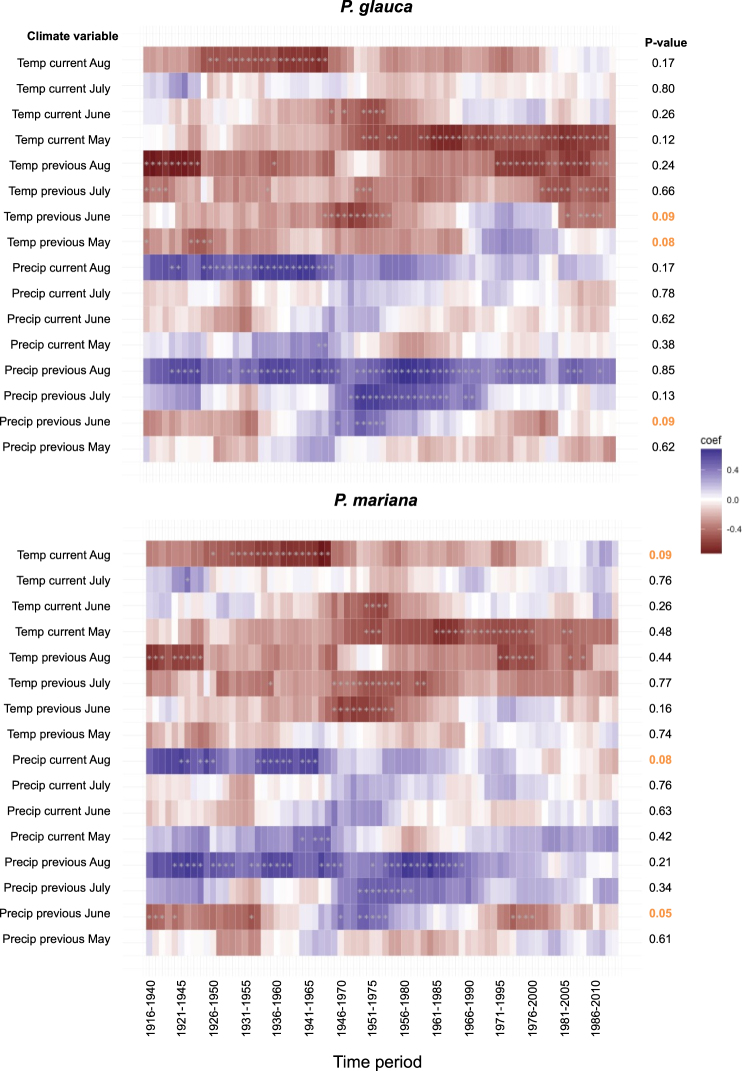
Moving window correlations between the black and white spruce ring width indices and growing season air temperature and precipitation. Blue shading indicates a positive correlation, red shading indicates a negative correlation and an asterisk indicates a statistically significant correlation. The P-values refer to a test of whether low frequency variation in the strength and sign of correlations over time is greater than would be expected by random chance (Zang & Biondi, 2015).

There was limited statistical evidence that climate-growth correlations have changed over the past century for either species (Fig. 3). Warmer growing seasons have been associated with reduced growth, while more abundant August precipitation has been associated with greater growth throughout the Fairbanks climate record. One possible exception is the relationship between May air temperature and growth. Both species showed greater negative sensitivity to warm May air temperature in recent decades than in the early part of the Fairbanks climate record. However, this change in the May temperature effect was not greater than might be expected by random chance.

### Form of Relationships between Climate and Tree Growth

**Figure 4:**
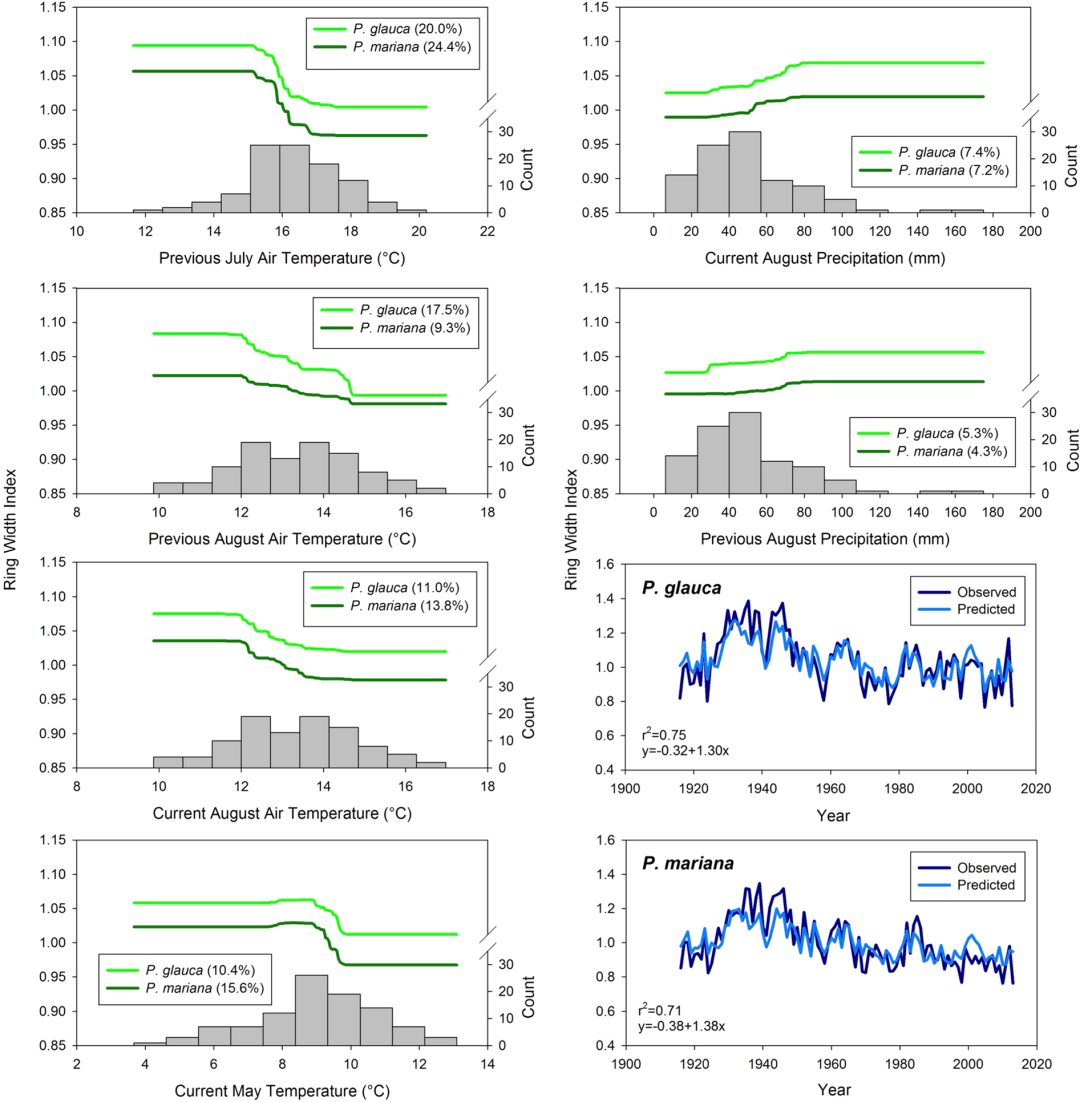
Partial dependence plots from BRT analyses designed to examine the form of relationships between the ring width indices and growing season air temperature and precipitation. The relative influence of each variable for each species is shown in parentheses, while the distribution of the climate data is shown by vertical bars. The final two panels show the relationship between the BRT climate-growth model predictions and the observed data for each species. While there were many years with warm growing season air temperature that help to constrain the lower tails of the sigmoidal relationships between temperature and growth, there were relatively few years with cool air temperature. The upper tails of the sigmoids are, therefore, rather poorly constrained.

Our climate-growth BRT models explained 71% of the variation in our black spruce chronology and 75% of the variation in our white spruce chronology (Fig. 4). Both models tended to slightly underestimate periods of greater growth and overestimate periods of lesser growth. Relationships between climate and tree growth were non-linear and remarkably similar for both species. Relationships between growing season temperature and growth were negative and sigmoidal in form, with growth declines observed when monthly mean temperatures passed through apparent thresholds (Fig. 4). Growth declines were generally observed over a 2–3 °C range, with growth stabilizing at lower levels under warmer air temperatures. Growth of both species showed a saturating response to increasing August precipitation. Growth was limited when August precipitation in Fairbanks was less than 40 mm, increased with rising precipitation between 40 and 80 mm and was stable at a greater level when August precipitation exceeded 80 mm. There was little evidence of strong interactions among the climate variables, suggesting that effects of warm growing seasons and low precipitation on growth were additive and not synergistic.

### Changes in Gas Exchange Physiology over Time

Random Forest regression analyses designed to isolate the effects of time period and ring age on Δ^13^C revealed that ring age was a more important determinant of Δ^13^C than time period for both black (ring age importance: 211.2, time period importance: 45.3) and white spruce (ring age importance: 171.6, time period importance: 34.3). Δ^13^C decreased strongly until ring age reached 50–70 years (~0.5‰ in black and ~1.5‰ in white spruce) and subsequently increased very slowly with rising ring age (~0.25‰/century in both species) (Supplemental Figure S1).

**Figure 5:**
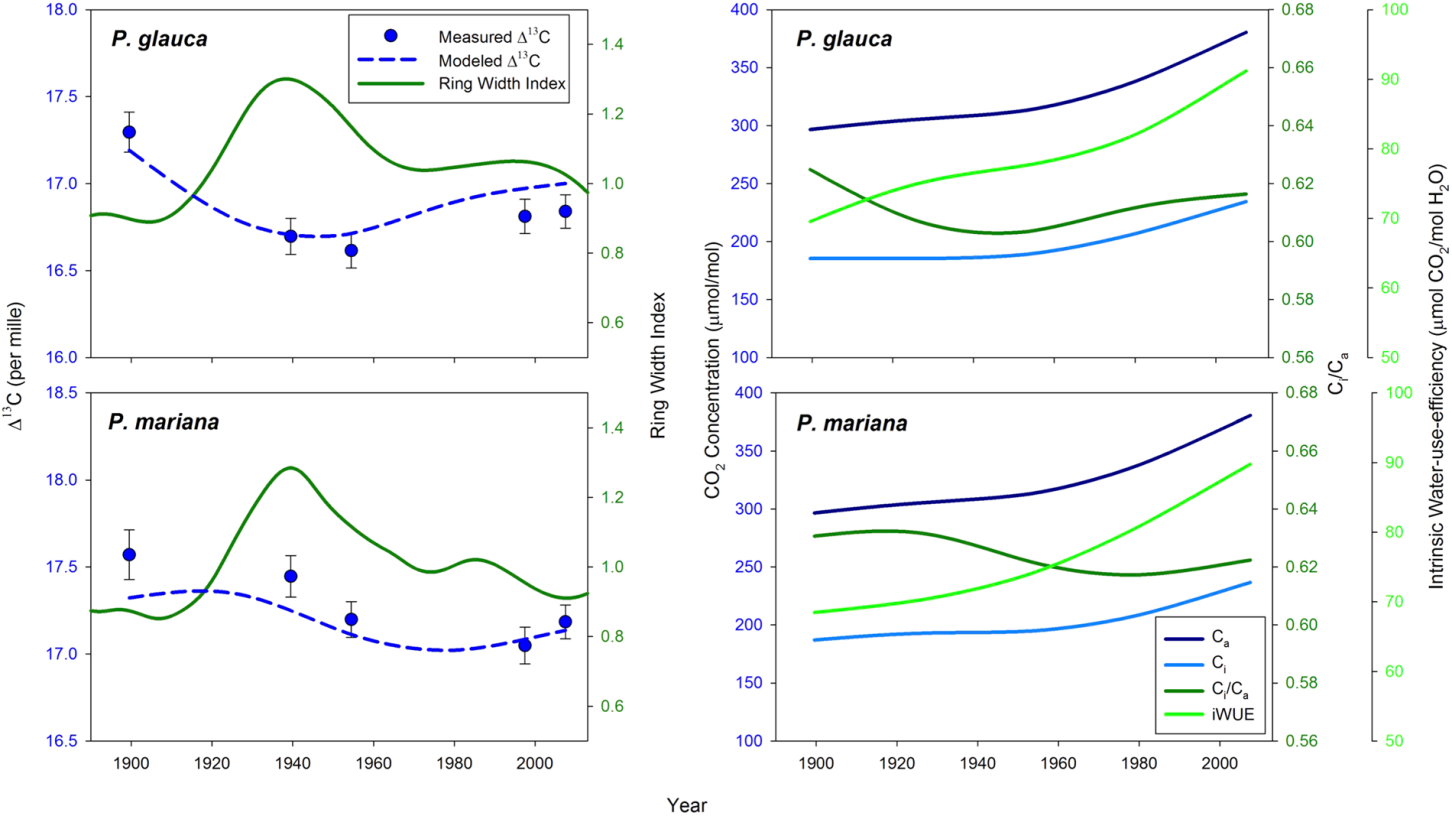
Measured and modeled Δ^13^C plotted in relation to smoothed four-curve RCS chronologies of black and white spruce growth. Bars on the measured data are standard error. The modeled data are partial dependence plots from a Random Forest analysis designed to separate the effects of time period and ring age and show Δ^13^C for a ring age of 100 years. The right-hand panels show atmospheric [CO_2_] (C_a_) (McCarroll & Loader, 2004), along with intercellular [CO_2_] (C_i_), C_i_/C_a_ and intrinsic water-use-efficiency (iWUE), which were calculated based on the modeled Δ^13^C estimates for each species.

In white spruce, the lowest Δ^13^C was observed during and immediately following the mid-20^th^ century growth peak, leading to an inverse relationship between growth and Δ^13^C (Fig. 5). For black spruce, the lowest Δ^13^C was observed during the 1993–2002 interval. Over the five time periods examined, C_i_/C_a_ varied by less than 0.03 in both species. In the context of rising C_a_, this relative constancy in C_i_/C_a_ was associated with a ~50 μmol/mol increase in C_i_ and a ~30% increase in iWUE of both species.

### Determinants of Spatial Variation in Recent Tree Growth

Our BRT models designed to explain spatial variation in recent growth (2003–2012) explained considerably less of the variation (23% for black and 39% for white spruce) than our models aimed at explaining temporal variation in the mean chronologies. In contrast with our temporal climate-growth analyses, which showed very small differences between species, our spatial analyses showed large differences in the habitat variables that most strongly influenced growth. For black spruce growth, the most influential variables were slope, aspect, moss cover, topographic position and stand age (Table 1), with limited evidence of strong interactions among predictors. Black spruce growth was greatest where the slope exceeded 15%, the aspect was not east-facing or flat ground, where moss cover was greater than 60%, on upper and mid-slopes and where the stand was younger (Supplemental Figure S2). For white spruce growth, the most influential variables were moss cover, duff depth, stand density and aspect. Recent white spruce growth was greater where moss cover was less than 50%, duff depth was shallow, stand density was higher and on flat ground and south-facing aspects. There was a strong interaction between duff depth and aspect. Increasing duff depth led to the greatest decline in growth on flat ground and south-facing aspects, where growth was greatest. Region, elevation and Δ^13^C had limited influence on spatial variation in recent growth of both species.

**Table 1:**
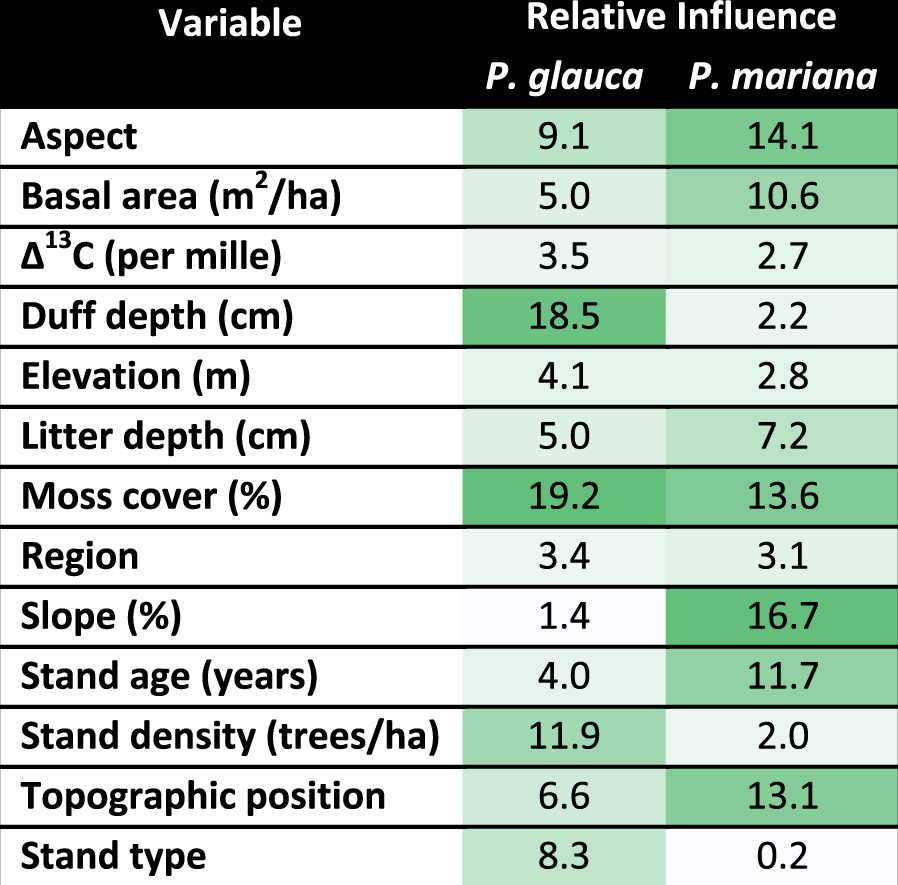
Relative influence of plot location variables, plot structural variables and gas exchange physiology on spatial variation in recent (2003–2012) growth of white and black spruce in interior Alaska. Darker shades of green indicate greater, while lighter shades indicate lesser influence. Relative influence sums to 100 for each species.

**Figure 6:**
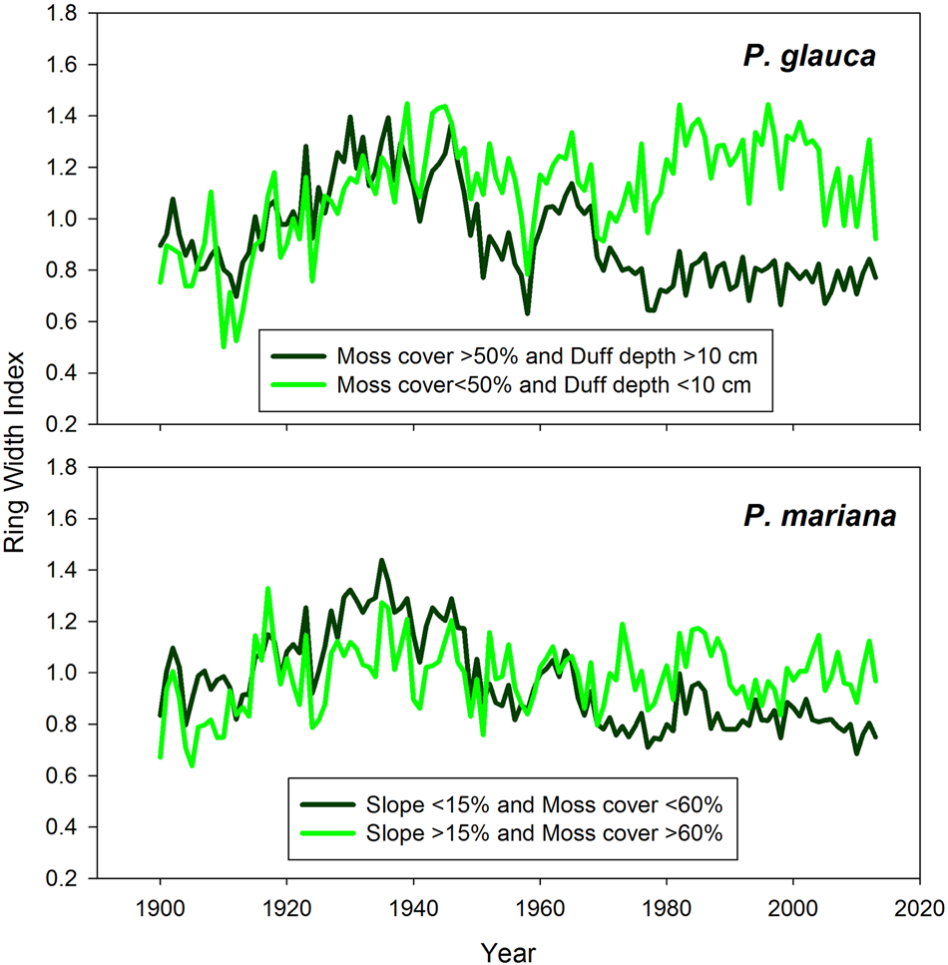
Chronologies of black and white spruce growing in good (light green) and poor habitats (dark green) during the 20^th^ century. Habitat quality was determined by examining partial dependence plots from a BRT analysis relating plot locational and structural variables to recent growth of both species (Supporting Figure S4). Sample sizes for white spruce are 86 trees from good habitats and 44 trees from poor habitats, while those for black spruce are 41 trees from good habitats and 53 trees from poor habitats. The trees and habitats selected represent the extremes of the population in terms of recent growth.

Construction of separate chronologies for black and white spruce growing in good and poor habitats revealed strong differences following the mid-20^th^ century growth peak (Fig. 6). When growing in poor habitat, both species showed clear evidence of the growth peak, followed by a decline to relatively stable growth from ~1970 to 2013. Black spruce growing in poor habitat may have shown a very slight decreasing trend following the growth peak, through 2013. When growing in good habitat, the mid-20^th^ century growth peak was less apparent for both species. Following an increase from ~1900 to ~1940, white spruce in good habitat generally maintained consistent growth through 2013. Meanwhile, black spruce growing in good habitat showed a slight increase during the early years of the 20^th^ century with very little trend thereafter.

Comparison of climate-growth correlations among black and white spruce growing in good and poor habitats revealed strong differences in temperature sensitivity (Table 2). Both species showed much stronger negative sensitivity to growing season air temperature when growing in poor habitats. Interestingly, white spruce growing in good habitat showed greater positive sensitivity to August precipitation than those growing in poor habitats. The opposite was true for black spruce, as trees growing in poor habitats generally showed greater positive sensitivity to growing season precipitation.

**Table 2:** Correlations between monthly climate data and ring width indices of black and white spruce growing in good and poor habitats (1916–2013).

Variable	Month	*P*. *glauca*		*P*. *mariana*	
		Good habitat	Poor habitat	Good habitat	Poor habitat
Precipitation	Previous May	0.009	0.106	0.003	0.107
	Previous June	0.033	−0.026	−0.109	−0.040
	Previous July	0.194	0.034	0.171	0.089
	Previous August	**0**.**394**	**0**.**291**	**0**.**263**	**0**.**261**
	May	0.070	0.154	0.145	**0**.**225**
	June	0.056	−0.028	0.007	−0.020
	July	−0.028	−0.075	−0.044	−0.036
	August	**0**.**278**	**0**.**254**	0.129	**0**.**229**
Air temperature	Previous May	0.035	**−0**.**443**	−0.131	**−0**.**392**
	Previous June	−0.060	**−0**.**392**	−0.189	**−0**.**377**
	Previous July	−0.150	**−0**.**547**	**−0**.**241**	**−0**.**546**
	Previous August	**−0**.**303**	**−0**.**444**	−0.200	**−0**.**398**
	May	−0.115	**−0**.**450**	**−0**.**281**	**−0**.**464**
	June	−0.044	−0.344	−0.121	−0.347
	July	0.133	−0.389	0.056	−0.428
	August	−0.186	**−0**.**362**	−0.133	**−0**.**386**

Habitat quality was determined by visually examining partial dependence plots from a BRT analysis relating plot locational and structural variables to recent growth of both species (Supporting Figure S3). Good white spruce habitat was defined as plots with less than 50% moss cover and a duff depth of less than 10 cm, while poor white spruce habitat was defined as plots with greater than 50% moss cover and duff deeper than 10 cm. Good black spruce habitat was defined as plots with a slope greater than 15% and moss cover greater than 60%, while poor black spruce habitat was defined as plots with less than a 15% slope and less than 60% moss cover.

## Discussion

Numerous studies have concluded that growth of black and white spruce in boreal Alaska is declining as a result of temperature-induced drought stress. The results of our study point to more nuanced responses of spruce growth to changes in climate. Our data confirm that growth of black and white spruce in the Tanana Valley is sensitive to moisture availability^6,8–12,27,28^, as evidenced by negative correlations with growing season air temperature, positive correlations with August precipitation and slight decreases in Δ^13^C over the past century. Fewer studies have examined the temporal stability of climate-growth relationships. Those that have^29^ also found that warm growing seasons and low August precipitation have been associated with limited growth of black and white spruce throughout the ~100 year Fairbanks climate record. The form of relationships between growth and growing season air temperature (sigmoidal) and August precipitation (saturating) also agree very well with results of an earlier study with a similar spatial extent^13^.

Our results differ from past work in several important respects. Many previous studies have produced tree-ring chronologies that show a steep growth decline during the latter half of the 20^th^ century^6,8,9,11,14^. These studies either did not detrend their ring width data^6,8^, detrended the majority of their tree-ring series by fitting a horizontal line^9,11^ or focused their analysis on just the latter portion of the 20^th^ century^10,14^. When exclusively old trees of similar age are sampled^6,8^, choosing *not* to detrend the data may allow the common age-related ring width decline to dominate the resulting tree-ring chronology. A similar effect can occur when trees of a wider age range are sampled, but tree-ring series are detrended by fitting a horizontal line, which eliminates variation in mean ring width across trees and allows the age-related ring width declines of both old and young trees to dominate the chronology^25^. Finally, while restricting the length of the chronology can be a means to avoid artifacts of the detrending process, doing so limits the opportunity to place recent growth in a historical context. Our black and white spruce chronologies both show a distinct growth peak near the middle of the 20^th^ century, followed by a decline to relatively stable growth in recent decades. Decline from a peak may have very different ecological consequences than decline from a trough.

Relationships between growing season air temperature and growth were sigmoidal, rather than linear, suggesting that growth may stabilize at a lower level at the warm end of the observed temperature range. While the sigmoidal relationships between growing season air temperature and growth of both species are well constrained at higher temperatures, they are poorly constrained at cooler temperatures, as there were relatively few years in Fairbanks with mean July air temperature <14 °C, for instance, during the 20^th^ century. We hypothesize that more limited growth of both species during the 19^th^ century was associated with generally cooler air temperature than observed during the 20^th^ century. Further, we anticipate that incorporating 19^th^ century climate data into our analyses (if they were available) would reveal temperature optima for growth at the lower end of the air temperature range observed during the 20^th^ century. While the flattening of the air temperature-growth relationships is well constrained at the warm end, it would not be wise to extrapolate beyond the air temperature range observed over the past century. Our results apply only to the range of climate conditions observed over the past century.

Several previous studies have examined Δ^13^C in rings of black and white spruce. One study restricted analysis of Δ^13^C in whole wood of black spruce to a ~25 year period (1979–2003) and found no evidence of a trend over time, although Δ^13^C was negatively correlated with growing season air temperature and positively correlated with growing season precipitation^14^. Another study showed overall variation of ~3.0‰ in white spruce holocellulose during the 20^th^ century and a decline of more than ~2.0‰ from ~1930 to 1996^6^. A third study showed a ~2.0‰ decline in Δ^13^C of black spruce holocellulose from ~1915 to 1996^8^. These dramatic declines in Δ^13^C over time dwarf the overall changes of <0.5‰ observed in our study. The difference between our results and these earlier studies may reflect contrasting sampling designs, differences in sample size and/or geographic extent and handling of potential age-related effects, particularly for rings formed during the first 50–70 years after a tree reaches breast height.

The very small overall changes that we observed in Δ^13^C during the past century are consistent with the set-point theory, which predicts that plants will respond to rising atmospheric [CO_2_] by reducing stomatal conductance to maintain constant C_i_/C_a_^30^. An earlier study in the northern boreal forest of Eurasia found very little change in C_i_/C_a_ of *Larix* and *Pinus* between the periods 1861–1890 and 1961–1990^31^. In their study, *Picea* showed a tendency toward lower C_i_/C_a_ in the more recent period at some sites. Our *Picea* data from interior Alaska and recent data from the Brooks Range^32^ are consistent with results for *Larix* and *Pinus* and suggest that maintenance of constant C_i_/C_a_ in the context of rising atmospheric [CO_2_] and associated changes in climate may be a common response of boreal trees at a global scale. Maintenance of near constant C_i_/C_a_ in the context of rising atmospheric [CO_2_] over the past century is associated with a ~50 μmol/mol increase in intercellular [CO_2_] and a ~30% increase in water-use-efficiency. Greater iWUE has important implications for the cycling of water and energy in boreal forests, as it is likely associated with reduced transpiration, more limited latent heat flux and a warmer and drier boundary layer.

In our study, growth of black and white spruce showed remarkably similar responses to climate, but very different effects of habitat on spatial variation in recent growth. Black spruce were most productive when growing on a slope and when moss cover was greater than 60%. Meanwhile, white spruce were most productive where moss cover was less than 50% and when duff depth was shallow. Moss cover and duff depth are likely integrator variables that reflect underlying site conditions and may not affect tree growth directly. For example, moss cover and duff depth are likely correlated with soil temperature, soil moisture and/or depth to permafrost and it may be these other variables that more directly affect tree growth. We hypothesize that greater black spruce growth may be associated with cool, moist soils without near-surface permafrost, while greater white spruce growth may be associated with relatively warm soils.

Separate chronologies created for black and white spruce growing in good and poor habitats revealed areas of the landscape where white spruce have sustained the relatively vigorous growth observed during the mid-20^th^ century growth peak. Meanwhile, black spruce growing on relatively flat ground with <60% moss cover in recent years showed greater negative sensitivity to temperature and slightly more positive sensitivity to growing season precipitation than other combinations of species and habitats. When Δ^13^C was examined separately for black spruce growing in good and poor habitats, two important differences emerged (Supplemental Figure S3). First, Δ^13^C was lower in black spruce growing in poor habitats, despite their lower mean elevation (Δ^13^C tends to decrease with increasing elevation)^33^. Second, there was evidence of a ~1.0‰ decline in Δ^13^C of black spruce growing in good habitats, but there was no evidence of a change in Δ^13^C of black spruce growing in poor habitats over the past century. These results are similar to observations in New Mexico, where lower elevation ponderosa pine that died in response to prolonged drought showed little variation in Δ^13^C during the years preceding mortality, while the higher elevation trees that survived showed strong decreases in Δ^13^C^34^. The authors concluded that the lower elevation trees were moisture limited prior to the onset of drought conditions and had a limited range over which to adjust foliar gas exchange, while higher elevation trees were able to reduce G_s_ in response to drought. It is somewhat counterintuitive that black spruce growing in flat or gently sloping areas might be the most sensitive to drought. When these habitats occur in upland areas, they are typically characterized by near surface permafrost that impedes drainage and leads to relatively high soil water content. However, several studies of white spruce further north in the Brooks Range have shown that G_s_ is reduced under low soil temperature^19,35^, presumably as a result of limited membrane permeability and greater water viscosity. Thus, it is possible the combination of high evaporative demand and cold permafrost-affected soils leads to conditions that are more chronically stressful than the coincidence of high evaporative demand and low soil water availability during the late summer in better drained hill slope habitats. In contrast, flat or gently sloping habitats that occur in lowland areas often exhibit patchy permafrost and sandy, well-drained soils, particularly in areas closer to the Alaska Range^36^. In these habitats, black spruce growth may be responding to both soil and atmospheric drought during warm and dry summers.

Some investigators have suggested that drought stress is leading to a decline in growth of black and white spruce, that widespread spruce mortality is imminent and that the boreal forest of interior Alaska is in the early stages of a shift in dominance from coniferous forests to temperate forests and/or grasslands^8,11^. Our data support a more nuanced interpretation that is generally more consistent with recent work in nearby Denali National Park and Preserve, where researchers have concluded that habitat diversity will likely provide resistance to a widespread transition in dominant vegetation type^37,38^ and with dynamic global vegetation models, which generally project greater boreal tree growth in a warmer climate^39^. Our results confirm that growth of black and white spruce in interior Alaska is sensitive to moisture availability, but also show that warm growing seasons with low August precipitation have been associated with limited spruce growth throughout the Fairbanks climate record. Overall, Δ^13^C in tree-ring alpha-cellulose showed limited variation over the past century, consistent with observations from across northern Eurasia and with the prediction that plants will respond to rising atmospheric CO_2_ by reducing stomatal conductance to maintain constant C_i_/C_a_. Collectively, our results beg the question: if spruce growth in interior Alaska is sensitive to moisture availability and the climate is becoming warmer and presumably drier, why has spruce growth not declined to historically low levels? We hypothesize that rising atmospheric [CO_2_] has been associated with a decrease in G_s_ that has left tree water use, photosynthetic CO_2_ uptake and growth of black and white spruce relatively unchanged, despite increasing evaporative demand. We stress that this is merely a hypothesis, but one that would benefit from testing with more detailed studies of tree physiological responses to changes in climate and atmospheric [CO_2_] in the boreal forest.

There are several limitations to our study. First, our results do not address the possibility that increasing wildfire frequency and severity may promote greater dominance by deciduous tree species at the expense of spruce in boreal Alaska^40^. Second, while isohydric species like spruce are expected to exhibit a prolonged growth decline prior to drought-induced mortality, episodic mortality events remain a possibility. Synergistic effects of unfavorable climate conditions and insect outbreaks like the spruce bark beetle outbreak of the late 1990s in southcentral Alaska^41,42^ could lead to episodic mortality events that would be difficult to predict based upon growth trends. Third, like all tree-ring studies, our results apply to only one aspect of growth (radial growth in the main stem), yet trees are well known to shift allocation patterns in response to changes in climate^43^. In that context, our results might not conflict with observations of decreasing landscape greenness in interior Alaska^3^. It is possible, if not likely, that black and white spruce of interior Alaska have shifted allocation from foliage to fine roots in response to increasing evaporative demand, while maintaining radial growth in the main stem near the long-term mean. Warming-induced deepening of the active layer may have facilitated greater belowground investment and the combination of deeper thaw and larger root systems may be another reason why spruce growth has not declined to a greater degree. Finally, like most tree-ring studies, our inferences are limited to individual trees that were alive when sampled and do not address changes over time in stand productivity. It is possible that mortality within the black and white spruce populations improved resource availability to surviving trees, thereby preventing growth declines. On the basis of our results and the limits to our inferences, we argue that more detailed studies of tree physiological responses to climate and atmospheric [CO_2_] that account for growth in all major organs and address changes in stand-level productivity are desperately needed in interior Alaska and throughout the boreal forest.

## Methods

### Increment Core Collection and Processing

Increment cores (1 core/tree) were collected at breast height (1.37 m) during the 2013 and 2014 growing seasons from United States Forest Service Forest Inventory and Analysis (FIA) and Alaska Integrated Resource Inventory System (AIRIS) plots in the Tanana Valley of interior Alaska (Fig. 1). FIA plots were restricted to forested land within the Tanana Valley State Forest (~730,000 ha) and Tetlin National Wildlife Refuge (~280,000 ha), while the AIRIS plots were more widely distributed. Approximately 5 cores/species were collected from each FIA plot (n = 100 plots), while approximately 10 cores/species were collected from each AIRIS plot (n = 9 plots), when the species was present on the plot. On the FIA plots, cores were collected from trees that appeared healthy and were representative of the most abundant size class. On the AIRIS plots, tree size cohorts were visually defined and cores were collected from representative trees within each cohort, regardless of apparent health. Our dataset contains a bias favoring visibly healthy trees that is lessened by incorporation of cores collected on the AIRIS plots^44^. Greater detail regarding the plots and the trees selected for sampling can be found in our earlier publication^25^.

Increment cores were air dried, mounted, sanded to 600 grit and measured to the nearest 0.001 mm using a sliding bench micrometer and digital encoder (Velmex Inc. Bloomfield, NY). Ring width data were analyzed in COFECHA to identify potential dating errors^45^. The pith was present in 27% of the white spruce and 40% of the black spruce cores that were included in the dataset. For cores that missed the pith, but passed close enough for the innermost ring to form a complete arc, the missing radius was estimated using the geometric method^46^. The number of missing rings was estimated by dividing the mean width of the first ten rings into the missing radius. Increment cores that did not include the pith or pass close enough for the innermost ring to form a complete arc were eliminated from the dataset. Additionally, 14 of the FIA plots sampled are known to have burned within the past 50 years. To limit the effect of disturbance on our tree-ring chronologies, we eliminated all post-fire ring width data from plots with a known fire history. A total of 339 white spruce and 213 black spruce cores (trees) were used to construct our tree-ring chronologies.

### Detrending

Ring width data were detrended using four-curve regional curve standardization (RCS) in CRUST^47^. We evaluated a wide range of common detrending methods and concluded that our four-curve RCS chronologies were the most unbiased and best correlated with the climate data of the methods tested^25^. Multiple curve RCS involves grouping the individual tree-ring series by mean ring width (>40 series/group), fitting an empirical curve describing the age-related ring width decline for each group and then calculating indices of observed versus expected ring width as a function of age. We calculated ring width indices as ratios of observed to expected growth and assembled chronologies using Tukey’s biweight robust mean. Data were processed to produce signal-free chronologies^48^, in which the raw ring widths are repeatedly divided by the detrended chronology (<10 iterations) with the goal of correcting for inadvertent removal of some of the climate signal during detrending. We truncated our chronologies when the sample size dropped below 50 trees.

Contrasting sampling sampling design between the FIA and AIRIS plots could lead to differences in growth trends. It would be reasonable to hypothesize that trees sampled on AIRIS plots might show greater evidence of declining growth in recent decades, as both apparently healthy and unhealthy trees were sampled. To examine this possibility, we produced separate multiple curve RCS chronologies for each species and plot type when data for at least 25 trees contributed to the mean (Supplemental Figure S4). For both species, inter-annual and inter-decadal variation tended to be greater in trees sampled on the AIRIS plots, although this could be attributable to smaller sample sizes. There was no evidence of a greater growth decline in recent decades among trees sampled on the AIRIS plots. On the contrary, white spruce sampled on the AIRIS plots showed a strong positive growth trend since the mid-1970s. Given the nature of the differences between the FIA and AIRIS tree-ring chronologies, we elected to conduct our analyses without regard to plot type.

### Potential Changes In Climate-Growth Relationships Over Time

Air temperature and precipitation data for Fairbanks, AK (1915–2013) were obtained from the Alaska Climate Research Center at the University of Alaska Fairbanks. We considered the possibility of using gridded and downscaled climate data in an effort to incorporate differences in climate means and trends across the Tanana Valley, but we were concerned about temporal heterogeneities associated with differences in station record lengths in those data sources^49^. Comparison of the Fairbanks growing season air temperature record with the records from five other climate stations in the interior boreal forest of Alaska and western Canada revealed good agreement in terms of inter-annual variability and long-term trends (Supplemental Figure S5).

In an earlier study using the same datasets^25^, we examined the strength and sign of correlations between our four-curve RCS chronologies and monthly mean air temperature (May-August) and both monthly and seasonal precipitation totals (May-August and October-April) for the full length of the Fairbanks climate record. These analyses were performed using the treeclim package^50^ in R 3.1.2^51^ and included climate data for the growth year and the previous year. Here, we extended our earlier analyses to examine potential changes in the strength and sign of climate-growth correlations over time for each species using moving window analyses in treeclim. The same climate variables used in the earlier static analyses were retained for the moving window analyses. Precipitation data were log-transformed prior to analysis, because they were positively skewed. We used a 25-year moving window beginning in 1916 with a 1-year step between windows. After completing the moving window analyses, we tested whether low-frequency variations in the strength and sign of correlations between climate and growth are significantly stronger than would be expected by chance, with significance assessed using exact bootstrap resampling.

### Form of Relationships between Climate and Tree Growth

The static and moving window correlation analyses are limited in the sense that they assume linear and non-interactive relationships between climate and tree growth. To examine the potential for non-linear relationships and interactions among climate variables, we conducted boosted regression tree (BRT) analyses^52^ separately for each species using the gbm^53^ and dismo^54^ packages in R 3.1.2. The same climate variables used in the moving window analyses were included in the BRT analyses. We used a tree complexity of 2, a learning rate of 0.001, a bag fraction of 0.5 and we set the maximum number of regression trees at 30000^13^. The final models were constructed using 3850 trees for white spruce and 3575 trees for black spruce. We examined the potential for interactions among climate variables and constructed partial dependence plots, which depict the modeled relationship between each climate variable and the ring width indices for each species, while holding all other variables at their mean values.

### Changes in Gas Exchange Physiology over Time

Carbon isotope discrimination (Δ^13^C) in tree-ring alpha-cellulose was examined to provide insight into potential changes in gas exchange physiology of black and white spruce over time. Following examination of the tree-ring chronologies, five time periods of interest were identified: 1895–1904, which was a period of relatively low and stable growth, 1930–1949, when growth of both species rose to a distinct peak, 1950–1959, when growth of both species declined from the peak, 1993–2002, which was a period of recent relatively stable growth and 2003–2012, which includes one of the warmest, driest and most severe wildfire seasons in recorded history (2004). A total of 85 black and 85 white spruce trees were selected for isotopic analysis with the aim of maximizing the inter-series correlation of the selected trees, obtaining an even distribution of both species across the study area and sampling similar numbers of old and young trees. The mean inter-series correlation of the selected trees was 0.420 for black and 0.503 for white spruce. Trees were selected for isotopic analysis on all but 26 of the 109 plots. The desire to sample young trees as well as old trees reduced the sample size for the 1895–1904 period to 53 black and 51 white spruce.

The time periods of interest were separated from each increment core and homogenized by slicing into fine fragments with a razor blade. The homogenized samples were then reduced to alpha-cellulose using the water-modified Brendel method^55,56^. The alpha-cellulose was dried overnight at 40 °C and 0.3 mg of each sample was weighed into a tin capsule for analysis using an elemental analyzer (Costech 4010, Costech Analytical, Valencia, CA), coupled with a continuous-flow isotope ratio mass spectrometer (Thermo-Finnigan Delta Plus XP, Thermo Electron Corp., Waltham, MA) in the Environment and Natural Resource Institute’s Stable Isotope Laboratory at the University of Alaska Anchorage. Carbon isotope discrimination (Δ^13^C) was calculated as

1 $${\rm{\Delta }}{}^{13}C=\frac{{\rm{\delta }}{}^{13}C_{a}-{\rm{\delta }}{}^{13}C_{tree}}{1+{\rm{\delta }}{}^{13}C_{tree}/1000},$$

where δ^13^C_a_ is the isotopic value of atmospheric CO_2_, which has decreased progressively as a result of fossil fuel combustion since the Industrial Revolution. Annual estimates of δ^13^C_a_ were obtained from the literature^57^. Data for 2003–2012 were estimated by linear extrapolation of the trend between 1993 and 2002.

Tree-ring Δ^13^C may be influenced by the age (size) of a tree. When a tree is young, it is likely to show greater Δ^13^C because it may assimilate a larger proportion of soil-respired CO_2_, because shade may lead to lower photosynthesis and/or because shorter trees exhibit lower resistance to xylem water flow, potentially allowing for greater stomatal conductance^58^. To address potential age effects on Δ^13^C, we conducted Random Forest regression analyses separately for each species with ring age and time period as independent variables and Δ^13^C as the dependent variable using the randomForest package^59^ in R 3.1.2. We then examined modeled Δ^13^C over time for each species with ring age held constant at 100 years, which was very close to the mean age of the trees selected for isotopic analysis.

To gain further insights into changes in gas exchange physiology over time, we calculated the ratio of intercellular to atmospheric [CO_2_] (C_i_/C_a_) from modeled Δ^13^C^60^:

2 $${C}_{i}/{C}_{a}=\frac{{\rm{\Delta }}{}^{13}{\rm{C}}-{\rm{a}}}{b-a},$$

where *a* is fractionation associated with diffusion of CO_2_ through the stomata (4.4‰), and *b* is fractionation during carboxylation (27‰). We then used annual estimates of C_a_^57^ to solve for C_i_. Again, estimates of C_a_ for 2003 to 2012 were obtained by linear extrapolation of the trend between 1993 and 2002. The relationship between C_i_/C_a_ and Δ^13^C was developed for whole leaf tissue, while our data are for tree-ring alpha-cellulose, which is enriched relative to whole wood and whole leaf tissue. To improve estimates of C_i_ and C_i_/C_a_, we applied an offset of −1.33‰ to δ^13^C of tree-ring alpha-cellulose^32,61^.

Finally, to further examine changes in the balance between photosynthesis (A) and stomatal conductance (G_s_) over time, we calculated intrinsic water-use efficiency (iWUE)^31^:

3 $${\rm{iWUE}}=\,\frac{A}{{G}_{s}}=({C}_{a}-{C}_{i})\ast \frac{1}{1.6}.$$

Carbon isotope discrimination in tree-rings is widely used to assess potential changes in moisture limitation to tree growth over time. Use of Δ^13^C in this context is complicated by two key factors. First, changes in Δ^13^C over time can be influenced by shifts in either A or G_s_, with changes in the former potentially masking or overriding changes in the latter. Second, there is a growing awareness that the Δ^13^C is actually related to the chloroplast CO_2_ concentration (C_c_), rather than C_i_, meaning that Δ^13^C is influenced both by G_s_ and by mesophyll conductance (G_m_)^62^. While G_m_ generally decreases with moisture limitation^63^ and most studies show a positive correlation between G_s_ and G_m_^64^, there may be instances when they are not well correlated and this may add uncertainty to interpretation of trends in Δ^13^C over time.

### Determinants of Spatial Variation in Recent Tree Growth

The FIA program collects a wide range of plot locational and structural variables. To investigate the most important drivers of spatial variation in recent growth of black and white spruce (2003–2012), we conducted BRT analyses similar to those used to examine relationships between climate and tree growth. Ring width indices were averaged for each tree over the 10-year period and related to the following variables: aspect (north, south, east, west), basal area (m^2^/ha), Δ^13^C (‰), duff depth (cm, the layer of decomposing organic matter below litter and above mineral soil), elevation (m), litter depth (cm), moss cover (%), region (northwest, northcentral, southcentral, southeast), slope (%), stand age (years), stand density (trees/ha), topographic position (upper slope, mid-slope, lower slope, alluvial flat, dry flat, wet flat) and stand type (black spruce, white spruce, paper birch (*Betula neoalaskana*), balsam poplar (*Populus balsamifera*), trembling aspen (*Populus tremuloides*)). The final models were constructed using 3925 regression trees for white spruce and 1090 trees for black spruce. Again, we examined the potential for interactions among plot variables and constructed partial dependence plots, which depict the modeled relationship between each variable and the ring width indices for each species, while holding all other plots variables at their mean values.

After identifying the two most influential plot locational or structural variables for each species, we examined partial dependence plots for obvious breaks or inflection points that would allow us to construct separate chronologies for black and white spruce growing in “good” and “poor” habitats. These separate chronologies represent the positive and negative extremes of our datasets with regard to recent growth. To examine the possibility that trees growing in good and poor habitats may respond to climate differently, we conducted static correlation analyses in treeclim using the same settings described above for the time period from 1916 to 2013. It is important to note that, while some plot variables are fixed (e.g., aspect, slope, elevation, etc.) others have likely varied through time (e.g., moss cover, duff depth, stand density, etc.). Our definitions of good and poor habitats relied on both fixed and temporally variable plot attributes. In the case of the latter, the underlying assumption is that recent moss cover, for instance, is a function of the edaphic characteristics of the plot, which have likely been more constant over time than moss cover itself.

### Data Availability

Data presented in this article have been submitted to the International Tree-Ring Data Bank for archival.

## Electronic supplementary material

Supplementary Information
