# Supplementary material for: Limited evidence of declining growth among moisture-limited black and white spruce in interior Alaska
Source: Sci Rep. 2017 Nov 10;7:15344. doi: 10.1038/s41598-017-15644-7 (PMC5681580; doi:10.1038/s41598-017-15644-7)

Title: Limited evidence of declining growth among moisture-limited black and white spruce in interior Alaska

Authors: Patrick F. Sullivan[1], Robert R. Pattison[2], Annalis H. Brownlee[1], Sean M.P. Cahoon[1] and Teresa N. Hollingsworth[3]

Affiliations: [1]Environment and Natural Resources Institute, University of Alaska Anchorage, Anchorage, AK, 99508, [2]Pacific Northwest Research Station, USDA Forest Service, Anchorage, AK 99503, [3]Boreal Cooperative Research Unit, USDA Forest Service, Fairbanks, AK 99775

Corresponding Author: Patrick F. Sullivan, University of Alaska Anchorage, 3151 Alumni Loop Rd., Anchorage, AK, 99508, 907-440-2865, pfsullivan@alaska.edu

Supplemental Figure S1. Partial dependence plots from a Random Forest analysis depicting the modeled effect of ring age on $\Delta^{13}C$, while holding calendar year constant at its mean value.

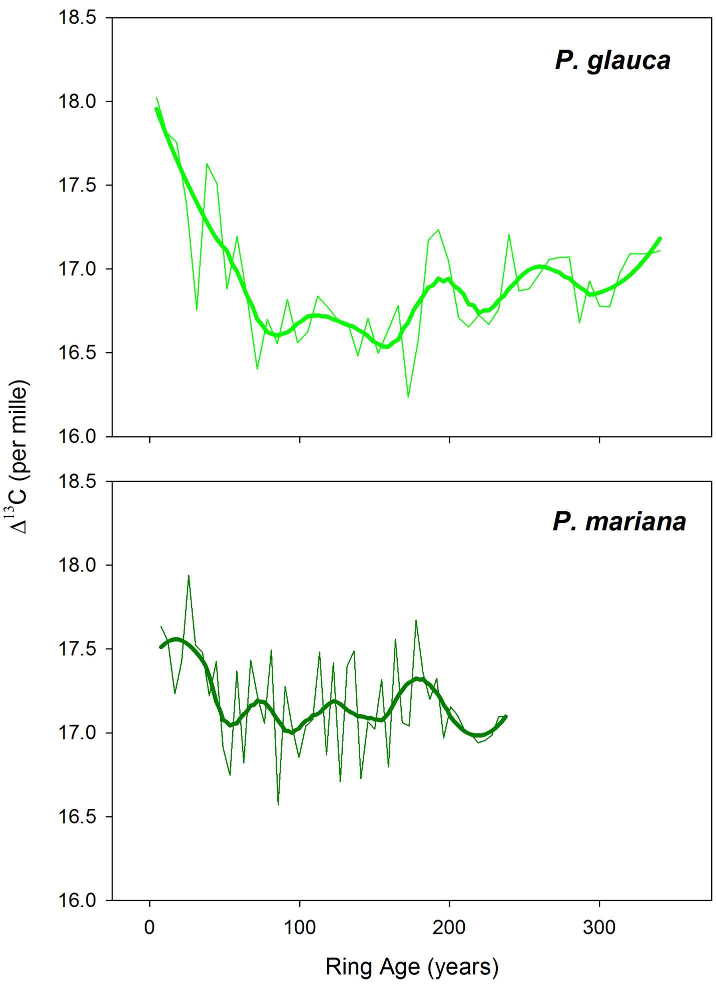

Supplemental Figure S2. Partial dependence plots from a Boosted Regression Tree analysis designed to identify the plot locational and structural variables that were the most important determinants of spatial variation in recent (2003-2012) growth of white and black spruce in interior Alaska. Slope and aspect were somewhat redundant predictors of recent black spruce growth, because low slope corresponds with no aspect. Slope and moss cover were used to define good and poor black spruce habitat in subsequent analyses, because of the overlap between slope and aspect and because sample sizes were small for black spruce growing on east, south and west aspects.

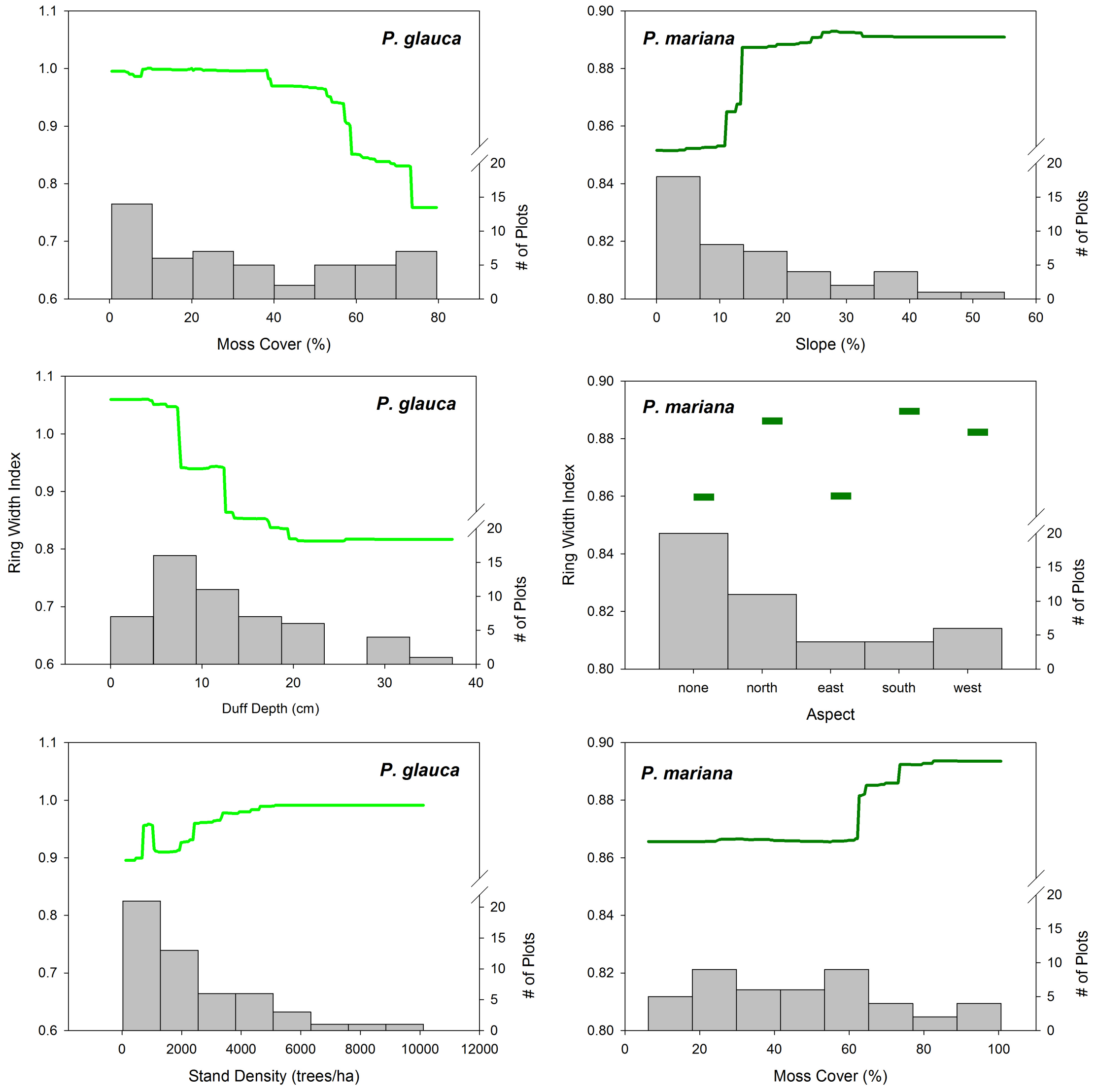

Supplemental Figure S3. Comparison of $\Delta^{13}C$ in tree-ring alpha-cellulose of black spruce growing in "good" (slope >15% and moss cover >60%) and "poor" habitats (slope <15% and moss cover <60%). The dashed lines show modeled data, derived from partial dependence plots of a Random Forest analysis designed to separate the effects of time period and ring age. The modeled data show $\Delta^{13}C$ for a ring age of 100 years. Comparison of $\Delta^{13}C$ in white spruce growing in good and poor habitats showed limited evidence of a difference.

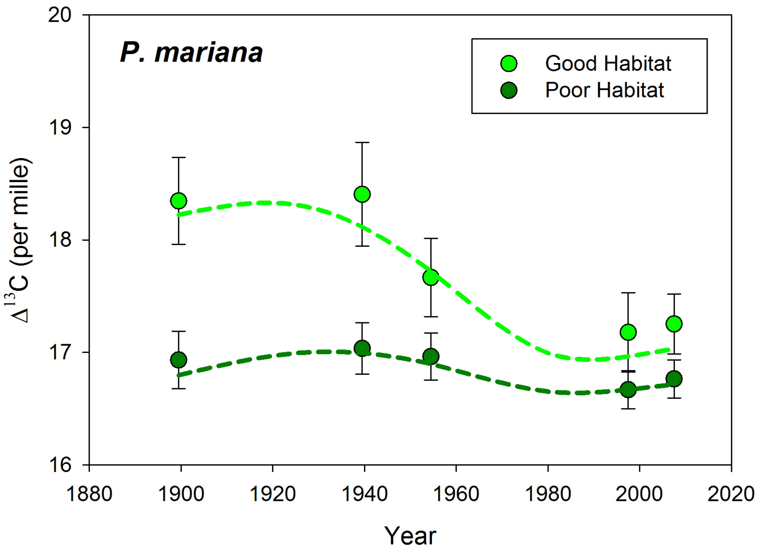

Supplemental Figure S4. Comparison of four-curve age RCS chronologies calculated separately for trees sampled on FIA and AIRIS plots when data for at least 25 trees were available to calculate the mean.

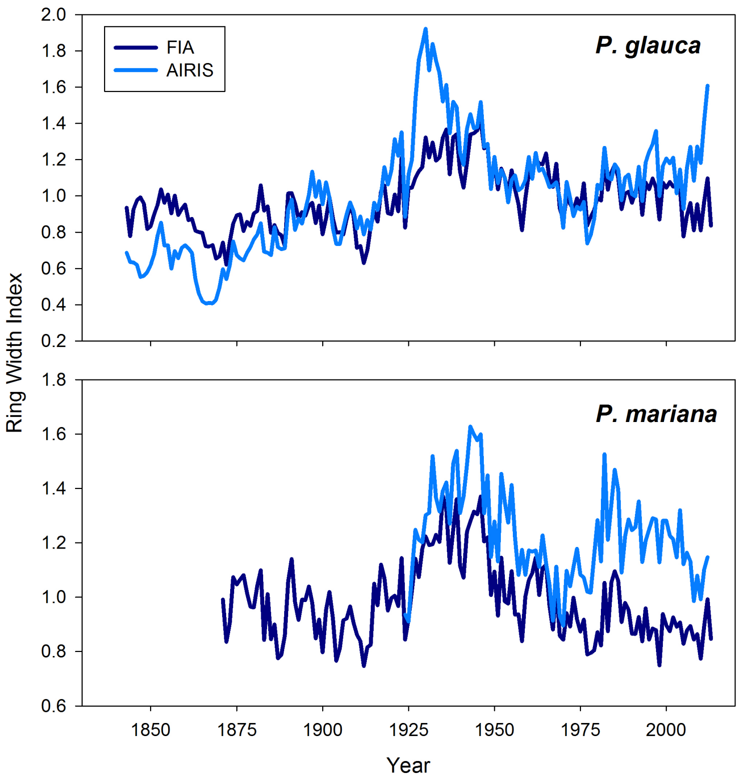

Supplemental Figure S5. Comparison of the Fairbanks, AK growing season air temperature record with records from five other stations in the interior boreal forest of Alaska and western Canada. Correlation coefficients are for the period common to all stations (1951-2008).

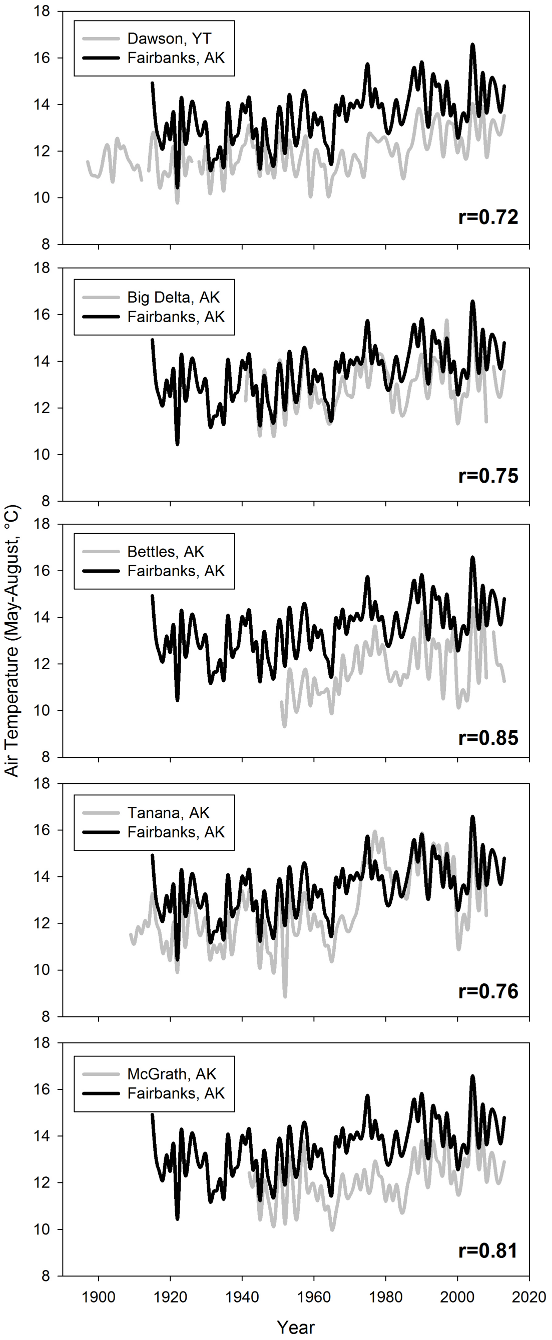

Supplement: Supplementary file 1 — Supplementary Information [file 41598_2017_15644_MOESM1_ESM.pdf]
